# Diagnostic and Prognostic Significance of microRNA-208a in Acute Myocardial Infarction

**DOI:** 10.1155/2022/7030722

**Published:** 2022-05-14

**Authors:** Sheng Chen, Xin Hong, Yong Wu, Ziguo Chen

**Affiliations:** ^1^Department of Cardiology, Affiliated Nanping First Hospital, Fujian Medical University, Nanping 353000, China; ^2^Xiamen Anxinya Biological Technology Co. Ltd., Xiamen, Fujian 361005, China

## Abstract

**Objective:**

To determine the prognostic and diagnostic significance of microRNA-208a (miR-208a) in acute myocardial infarction (AMI).

**Methods:**

Totally, 84 AMI patients hospitalized in our hospital between Jan. 2019 and Feb. 2021 were enrolled as the patient group (Pat group), and 50 healthy individuals over same time span as the control group (Con group). qRT-PCR assay was carried out to quantify serum miR-208a in the patients and receiver-operating characteristic (ROC) curves for analysing its diagnostic value in AMI patients and its predictive value in clinical efficacy and adverse events in the patients after therapy. The changes of miR-208a and clinical indexes ((lactate dehydrogenase (LDH), creatine kinase (CK) as well as Creatine kinase-MB (CK-MB)) in the patients before and after therapy were evaluated. Pearson's test was adopted to analyse the associations of miR-208a with clinical indexes. Additionally, the target genes of miR-208a were forecasted.

**Results:**

The patient group showed a higher miR-208a level than the control group (*p* < 0.05), and the area under the curve (AUC) of miR-208a in diagnosing AMI was >0.9. After therapy, patients presented notable decreases in serum miR-208a, LDH, CK, and CK-MB (all *p* < 0.05). Serum miR-208a presented positive associations with LDH, CK, as well as CK-MB both before and after therapy (all *p* < 0.05). Before therapy, the ineffective group presented a higher miR-208a level than the effective group (*p* < 0.05), and miR-208a had an AUC of 0.784 in forecasting efficacy. Additionally, the group with adverse events presented a higher miR-208a level than the group without them before therapy (*p* < 0.05), and miR-208a had an AUC of 0.713 in forecasting adverse events. According to enrichment analysis, the target genes of miR-208a were bound up with signal pathways of cellular senescence, MTOR and Wnt.

**Conclusion:**

With high expression in AMI cases, miR-208a is a promising potential biomarker for diagnosis and prognosis forecasting of AMI.

## 1. Introduction

Acute myocardial infarction (AMI) is a frequently seen serious cardiovascular disease with acute onset in clinical scenarios, belonging to one serious manifestation of coronary artery disease [1]. Because of the acceleration of the global aging process, the change of people's living habits, and the pollution of the surrounding environment, cardiovascular diseases present an annually growing incidence, especially AMI [2]. Statistically, approximately 1.5 million people suffer AMI each year in the United States. According to some surveys, AMI shows a growing mortality in both urban and rural population in China, with an increase of 5.6 times from 1987 to 2014, and 4%-15% of patients with it are younger than 45 years in recent years, showing that more young people is afflicted by it [3].

Clinically, AMI has no notable clinical manifestations at the initial phase, which can likely to result in missed diagnosis and misdiagnosis, so many patients come to see a doctor only when they have acute onset and thus miss the optimal therapy timing and suffer unfavourable prognosis [4]. At the current stage, the laboratory indexes frequently adopted for clinical evaluation of myocardial injury include creatine kinase isoenzyme (CK-MB), lactate dehydrogenase (LDH), and cardiac troponin (cT-nI/T), but they are limited due to their poor specificity and aberrant expression in other diseases [5]. Accordingly, the key to solving this problem lies in finding a novel diagnostic marker.

MiR is one noncoding RNA with an endogenous length between 20 and 25 nt [6]. According to one study [7], miR is able to interact with specific mRNAs by inducing the degradation of mRNAs or suppressing the translation, thus regulating the expression in diseases after transcription. An increasing number of studies have discovered the participation of miR in various basic biological processes, including proliferation, differentiation, necrosis, apoptosis, autophagy, development, and aging [8]. Reportedly, miR is abnormally expressed in diseases including cancer, cardiovascular diseases, and immunodeficiency diseases [9]. The most ideal diagnostic markers should be readily available and detectable. Interestingly, according to quite a number of reports, miR exists in serum and plasma in a consistent, stable, and repeatable way, which greatly arouses the interest of scholars in using miR in circulation as a biomarker [10].

MiR-208a, namely, miR-208a-3p, has been discovered to be with an association with the development of various cancers. For instance, Cui et al. [11] found that compared with miR-negative control (NC), miR-208a intensified the proliferation and invasion of gastric cancer cells through targeted secretion of SFRP1 and negative regulation on maternal expressed gene 3. MiR-208a acts as one oncogene in colorectal cancer via PDCD4 [12]. Moreover, reportedly, miR-208a aggravates CCl 4-induced liver injury in mice due to the lack of pathways activating cell death [13]. The association of miR-208a with AMI has been reported, but more research is needed to verify whether miR-208a can serve as a diagnostic marker of AMI.

In the present study, we analysed the diagnostic and prognostic significance of miR-208a in AMI and the potential mechanism of miR-208a in AMI to offer reference to clinical therapy and efficacy prediction.

## 2. Patients and Methods

### 2.1. Clinical Data

Totally, 84 AMI patients admitted to our hospital between January 2019 and February 2021 were enrolled into the patient group (Pat group), including 54 males and 30 females, between 48 and 80 years old, with a mean age of 63.9 ± 7.0 years, and 50 healthy individuals who underwent physical examination in our hospital during the same time span were enrolled as the control group (Con group), including 28 males and 22 females, between 43 and 78 years old, with a mean age of 60.3 ± 6.9 years. There was no significant difference exists between the two groups in gender and age (*p* > 0.05). This study was approved by the ethics committee of Affiliated Nanping First Hospital, Fujian Medical University. Signed written informed consents were obtained from all participants before the study.

### 2.2. Inclusion and Exclusion Criteria

The inclusion criteria are as follows: patients confirmed with acute myocardial infarction with ST-segment elevation for the first time at the first onset based on 2017 ESC Guidelines for the management of AMI in patients presenting with ST-segment elevation [14], patients who had undergone percutaneous coronary intervention (PCI), and those with detailed clinical data.

The exclusion criteria are as follows: patients with comorbid tumour or immune function diseases, patients with congenital organ defects, patients who were not suitable to receive the operation, patients intolerant of the drugs applicable to this operation, and those with mental disorder.

The inclusion and exclusion criteria of the control group are as follows: healthy individuals with normal serum indexes and imaging indexes were enrolled.

### 2.3. Therapeutic Regimen for the Patients

All patients were given PCI. Specifically, each patient was ordered to orally take 300 mg aspirin before PCI and orally take 600 mg clopidogrel at the beginning of the surgery, and the patient was also ordered to orally take clopidogrel at 150 mg/time, once a day, and aspirin at 100 mg/time, once a day, after surgery for 7 consecutive days.

### 2.4. qRT-PCR

Total mRNA in tumour cells was acquired via TRIzol reagent (Invitgen, Carlsad, CA, USA), and the extracted RNA was inversely transcribed into cDNA using SYBR PreMix Ex Taq Kit (TaKaRa Bio, Inc., Tokyo, Japan) under manual guidelines. The forward and reverse primers of miR-208a were 5′-CGCGGCATAAGACGAGCAAAAAGC-3′ and reverse 5′-ACGACAGTTCAACGGCAGCACCG-3′, respectively, and those of U6 were 5′-ATCGCCTTCGGCAGCACA-3′ and 5′-CACGCTGCACGAATTCGCGT-3′, respectively. qRT-PCR was conducted as follows: 95°C/5 min, 95°C/10 s, and 60°C/45 s, 40 cycles in total, and the expression data of miR-208a were normalized to U6 expression of the same sample. The Ct value of every target gene was normalized with that of the reference gene (Ct (Ct(miR-208a)-Ct(U6). The relative expression was calculated using the 2^−ΔΔCT^ method.

### 2.5. Detection of Clinical Indexes

Morning fasting venous blood (5 mL) was acquired from each patient via one coagulation-promoting tube, and 5 mL of it was acquired via one heparin sodium anticoagulation tube, all of which were treated by 10 min centrifugation (3,000 r/min) for separating serum/plasma. The changes of myocardial enzymes (LDH, CK, and CK-MB) were observed via one automatic biochemical analyzer (Beckman Coulter AU5800, Franklin Lakes, NJ, USA) before and after therapy.

### 2.6. Follow-Up

The patients were followed up for 6 months, once every 2 months, via telephone and reexamination of outpatient data to understand adverse cardiovascular events in them, including cardiogenic death, recurrent angina pectoris, nonfatal AMI, heart failure, and malignant arrhythmia.

### 2.7. Predication of Target Genes

The online databases (Targetscan, miRDB, and starBase) were utilized for forecasting the possible target genes of miR-208a, and the corresponding Wayne map was drawn.

### 2.8. Enrichment Analysis

GO and KEGG enrichment analyses were carried out using DAVID (6.8) online database. The former was conducted with three modules (biological process (BP), cellular component (CC), and molecular function (MF)) for functional annotation, and the latter can display the importance of different signal pathways in protein interaction network. Finally, the bubble charts of GO and KEGG enrichment analyses were drawn and visualized. The standards of |NES| > 1, *p* value < 0.05, and FDR < 0.25 were adopted.

### 2.9. Outcome Measures

Primary outcome measures are as follows: miR-208a in AMI patients was analysed, on which its value in diagnosing AMI was evaluated via ROC curves. The value of miR-208a in predicting clinical efficacy on patients was also analysed, and the evaluation criteria of efficacy was summarized in Table 1. The value of miR-208a in predicting the occurrence of adverse events after therapy was also evaluated.

Secondary observation indexes are as follows: the changes of miR-208a and clinical indexes before and after therapy were evaluated. The Pearson's test was adopted to analyse the associations of miR-208a with clinical indexes, and the target genes of miR-208a and bioinformatics were conducted for understanding the value of miR-208a in the diagnosis of AMI. The value of miR-208a in predicting clinical efficacy on patients was also analysed, and the evaluation criteria of efficacy was summarized in Table 1. The value of miR-208a in predicting the occurrence of adverse events after therapy was also evaluated.

### 2.10. Statistical Analyses

Statistical Product and Service Solutions (SPSS) 21.0 (IBM, Armonk, NY, USA) for data analysis and GraphPad Prism 8.0 (La Jolla, CA, USA) for figure drawing. The counting data were analysed via the *t*-test, and their intergroup comparison and intro-group comparison were carried out using the independent-samples *t*-test and paired *t*-test, respectively. Measurement data were analysed via the chi-square test, and ROC curves were drawn to predict efficacy and the occurrence of adverse events. *p* < 0.05 implies a notable difference.

## 3. Results

### 3.1. Expression and Diagnostic Significance of miR-208a in AMI

**Figure 1:**
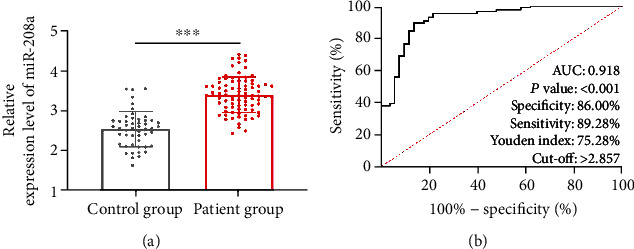
Expression and diagnostic significance of miR-208a in AMI. (a) Quantification of serum miR-208a in the control and patient groups via qRT-PCR. (b) Diagnostic value of miR-208a in AMI patients according to ROC curve. Note: ^∗∗∗^*p* < 0.001.

Serum miR-208a in the patients was quantified via qRT-PCR, and comparison of it between the two groups showed that the miR-208a level in the patient group was notably higher than in the control group (*p* < 0.05, Figure 1(a)). ROC curves were adopted to analyse the clinical value of miR-208a in distinguishing healthy individuals from AMI patients, and its area under the curve (AUC) was >0.9 (*p* < 0.05, Figure 1(b)), implying its great potential to be biomarker with high clinical value.

### 3.2. Changes of miR-208a and Clinical Indexes in Patients before and after Therapy

**Figure 2:**
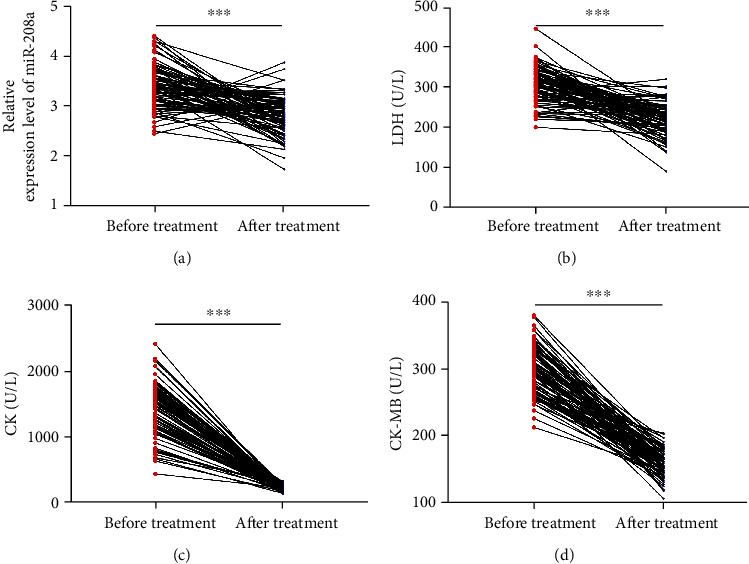
Changes of serum miR-208a, LDH, CK, and CK-MB in patients before and after therapy. (a) Changes of serum miR-208a in patients before and after therapy according to qRT-PCR assay. (b) Changes of serum LDH in patients before and after therapy according to the automatic biochemistry analyzer. (c) Changes of serum CK in patients before and after therapy according to the automatic biochemistry analyzer. (d) Changes of serum CK-MB in patients before and after therapy according to the automatic biochemistry analyzer.

For more deeply understanding the association of miR-208a with patients' condition, we analysed the changes of miR-208a before and after therapy and found a notable decrease in serum miR-208a in the patients after therapy (*p* < 0.05, Figure 2(a)). Additionally, after therapy, patients presented notable decreases in serum LDH, CK, and CK-MB (all *p* < 0.05, Figures 2(b)–2(d)).

### 3.3. Correlation Analysis of miR-208a with Clinical Indexes

**Figure 3:**
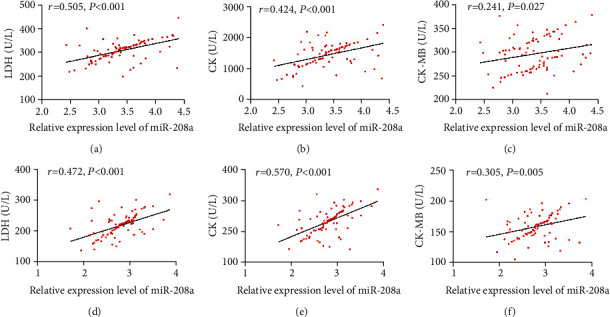
Correlation analysis of miR-208a with LDH, CK, and CK-MB before and after therapy. (a) Association of miR-208a with LDH before therapy according to the Pearson's test. (b) Association of miR-208a with CK before therapy according to the Pearson's test. (c) Association of miR-208a with CK-MB before therapy according to the Pearson's test. (d) Association of miR-208a with LDH after therapy according to the Pearson's test. (e) Association of miR-208a with CK after therapy according to the Pearson's test. (f) Association of miR-208a with CK-MB after therapy according to the Pearson's test.

For further analysing the associations of miR-208a with AMI development, the Pearson's Test was adopted for understanding the associations of miR-208a with LDH, CK, and CK-MB. The results revealed positive associations of miR-208a with them (all *p* < 0.05, Figure 3).

### 3.4. Expression and Predictive Value of miR-208a in Patients with Different Efficacy before Therapy

**Figure 4:**
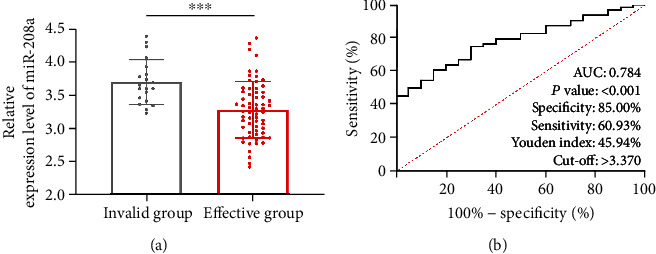
Value of miR-208a in forecasting the efficacy on AMI patients after PCI. (a) Serum miR-208a in patients with different efficacy according to qRT-PCR. (b) Predictive value of miR-208a in efficacy improvement of AMI patients after PCI based on ROC curve. Note: ^∗∗∗^*p* < 0.001.

After therapy, we evaluated the efficacy on the patients and found 26 patients with markedly effective efficacy, 38 patients with effective efficacy, and 20 patients with ineffective efficacy. The 26 patients and 38 patients were assigned to the improvement group (*n* = 64), and the rest were assigned to the nonimprovement group (*n* = 20). According to comparison of miR-208a between the two groups before therapy, the nonimprovement group presented notably higher miR-208a expression than the other group (*p* < 0.05, Figure 4(a)). According to ROC curve-based analysis, the AUC of miR-208a in predicting the improvement of clinical efficacy (*p* < 0.05, Figure 4(b)), suggesting the potential of miR-208a to be a biomarker for forecasting the efficacy improvement on AMI patients after PCI.

### 3.5. Predictive Value of miR-208a before Therapy for Postoperative Adverse Events in Patients

**Figure 5:**
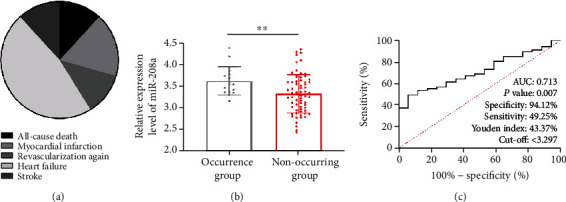
Predictive value of miR-208a in the occurrence of postoperative adverse events in patients. (a) Distribution of adverse events. (b) Serum miR-208a in patients with postoperative adverse events according to qRT-PCR. (c) Predictive value of miR-208a in AMI patients after PCI according to ROC curve. Note: ^∗∗^*p* < 0.001.

We also counted adverse events within 6 months after therapy. In the past six months, 17 patients suffered adverse events, including 2 cases with all-cause death, 3 cases with myocardial infarction, 2 cases with revascularization, 8 cases with heart failure, and 2 cases with stroke. The patients were assigned to two groups in light of the occurrence of adverse events: occurrence group and nonoccurrence group (Figure 5(a)). The occurrence group presented notably higher serum miR-208a than the other before therapy (*p* < 0.05, Figure 5(b)). According to ROC curve-based analysis, miR-208a had an AUC of 0.713 in forecasting the occurrence of adverse events (*p* < 0.05, Figure 5(c)), suggesting the potential of miR-208a to be a biomarker for forecasting them in AMI patients after therapy.

### 3.6. MiR-208a Target Gene and Bioinformatics Analysis

**Figure 6:**
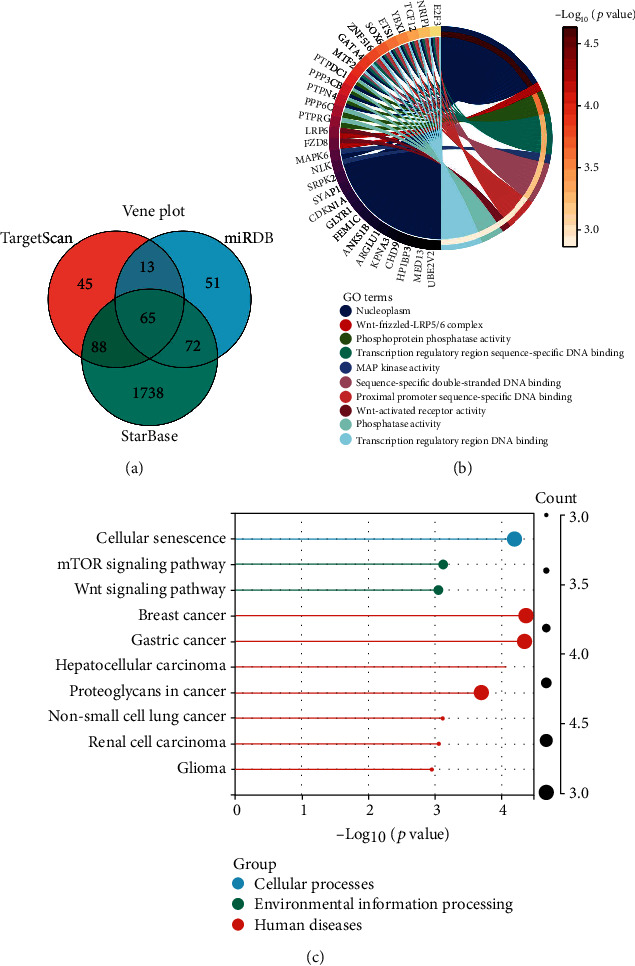
MiR-208a target gene and bioinformatics analyses. (a) Target genes of miR-208a predicted by Targetscan, miRDB, and starBase. (b) GO enrichment analysis of the functions of 65 common target genes of miR-208a. (c) KEGG enrichment analysis of 65 target genes involved in miR-208a pathway.

We predicated the target genes of miR-208a by Targetscan, miRDB, starBase, and found 211 target genes via Targetscan, 201 via miRDB and 1963 via starBase. Through the Wayne map, we found 65 common target genes (Figure 6(a)). Then, based on DAVID online software analysis, GO enrichment revealed the primary enrichment of miR-208a target genes in the functions of MAP kinase activity, Wnt-activated receptor activity, and phosphatase activity (Figure 6(b)), and KEGG enrichment analysis revealed the associations of miR-208a target genes with cellular senescence and MTOR and Wnt signalling pathways (Figure 6(c)).

## 4. Discussion

The primary pathogenesis of AMI is the occurrence of thrombosis triggered by the release of many platelets due to the rupture of coronary atherosclerotic plaque [15]. The continuous expansion of infarct area gives rise to coronary artery occlusion and slow blood flow, triggering systemic microcirculation disturbance and resulting in progressive left ventricular dilatation and deterioration of heart function [16]. Definitive diagnosis and timely guidance of clinical therapy are the key to improving the prognosis of patients with AMI.

A growing number of studies have revealed the participation of miR in the life processes of organism, its wide distribution in human body, and its expression in only specific cells and tissues [17]. MiR-208a, as one miR discovered early, is expressed in diseases including tumour and immune function diseases [18]. Feng et al. [19] have revealed the high expression of miR-208a in rat models of early myocardial infarction, suggesting that miR-208a has the potential to be a marker of AMI. In our study, serum miR-208a was quantified via qRT-PCR. The results showed its high expression in AMI patients and its high value in distinguishing AMI patients from healthy individuals (AUC > 0.9). Similar to our study results, Oyunbileg et al. [20] have also found the high expression of miR-208a in AMI patients and its AUC > 0.9. Our research once again verified the diagnostic value of miR-208a in AMI.

Prior research has confirmed the diagnostic significance of miR-208a in AMI, but whether miR-208a changes in AMI patients after therapy has not been studied and analyzed. Over the past few years, as people have increasingly understanding of clinical research and clinical therapy of AMI, especially the application of interventional therapy, there is a new therapeutic scheme for clinical therapy of AMI [21]. With increasingly extensive application in clinical practice, PCI is regarded as one effective method to relieve myocardial necrosis and increase coronary perfusion [22]. In our study, the changes of miR-208a after PCI were analysed. According to the results, after therapy, patients presented notably downregulated miR-208a. Correlation analysis revealed positive associations of miR-208a with myocardial function indexes before and after therapy, which suggested that miR-208a could serve as one observation index for AMI patients before and after therapy. For more deeply determining the association of miR-208a with efficacy on AMI patients, the patients were grouped in light of clinical efficacy after therapy. According to the results, the nonimprovement group presented a notably higher miR-208a level than the improvement group before therapy, suggesting the possible value of miR-208a in forecasting the improvement of efficacy on AMI patients. Thus, corresponding ROC curves were drawn, showing an AUC of miR-208a >0.7 in forecasting the clinical efficacy on AMI patients, which implied the potential of miR-208a in forecasting the clinical efficacy on AMI patients.

PCI has become the primary therapy for AMI, but it can only relieve the narrow lesion, and patients still face a risk of recurrent adverse cardiac events after it because of the persistence of risk factors leading to the disease continue [23]. Our study investigated the adverse events of patients for 6 months by telephone and outpatient medical records. Among the 84 patients, 17 patients suffered adverse events after therapy, showing an incidence of 20.23%. The patients were grouped in light of the adverse events, and the comparison revealed notably higher miR-208a expression in patients with AMI than in those without it, and an AUC > 0.7, which implied the function of miR-208a in AMI patients before therapy in serving as a predictor of adverse events.

Finally, the target genes of miR-208a were forecasted. As a result, 85 potential target genes were found. GO and KEGG enrichment analyses revealed the correlations of miR-208a with MTOR and Wnt signalling pathways. Wei et al. [24] have discovered the participation of autophagy in the protection against acute myocardial infarction via 1,25-dihydroxyvitamin D3 by PI3K/AKT/mTOR pathway. Another study has discovered the impact of miR-154 on cardiomyocyte apoptosis in rats with AMI via Wnt/*β*-catenin signalling pathway [25]. The results suggest the possible involvement of miR-208a in the occurrence of AMI through many ways and also pave a way for our follow-up research.

Our study has determined the diagnostic and predictive value of miR-208a in AMI patients, but it still has some limitations. First of all, we only collected the serum of healthy individuals and AMI patients, so the existence of difference in miR-208a expression between patients with unstable angina pectoris and AMI patients needs further study. Secondly, as a clinical study, the mechanism of miR-208a in AMI has yet to confirm. We only analysed its potential mechanism through the prediction of target genes, but whether miR-208a can participate in the occurrence of AMI by regulating these functions still needs experimental verification. Therefore, we hope to carry out more experiments in the future to improve our research.

All in all, with high expression in AMI cases, miR-208a is a promising potential biomarker for diagnosis and prognosis forecasting of AMI.

## Figures and Tables

**Table 1:** Clinical efficacy evaluation.

Efficacy grading	Evaluation criteria
Markedly effective	The symptoms of heart failure disappeared; the heart rate was less than 100 beats/min; the heart function was grade I.
Effective	The symptoms of heart failure were alleviated; the heart rate was more than 100 beats/min; the heart function grade was grade II-III.
Ineffective	Electrocardiogram showed no improvement or even showed aggravation in various indexes, clinical signs, and symptoms.

## Data Availability

The clinical data used to support the findings of this study are included within the article.
